# 2785. Characterization of Methicillin-resistant *Staphylococcus aureus* Bloodstream Isolates Recovered from Patients Enrolled in a Randomized, Double-blind, Multi-center Study to Establish the Efficacy and Safety of Ceftobiprole for Treatment of Bacteremia, Including Infective Endocarditis

**DOI:** 10.1093/ofid/ofad500.2396

**Published:** 2023-11-27

**Authors:** Rodrigo E Mendes, Leonard R Duncan, John H Kimbrough, Thomas L Holland, Vance G Fowler, Mark E Jones, Marc Engelhardt, Jennifer Smart, Mariana Castanheira

**Affiliations:** JMI Laboratories, North Liberty, Iowa; JMI Laboratories, North Liberty, Iowa; JMI Laboratories, North Liberty, Iowa; Duke University Medical Center, Durham, NC; Duke University Medical Center, Durham, NC; Basilea Pharmaceutica International Ltd., Allschwil, Switzerland, Allschwil, Basel-Landschaft, Switzerland; Basilea Pharmaceutica International Ltd., Allschwil, Switzerland, Allschwil, Basel-Landschaft, Switzerland; Basilea Pharmaceutica International Ltd, Allschwil, Basel-Landschaft, Switzerland; JMI Laboratories, North Liberty, Iowa

## Abstract

**Background:**

A Phase 3 clinical trial evaluated ceftobiprole (BPR) for the treatment of *Staphylococcus aureus* bacteremia (SAB), including right-sided infective endocarditis (NCT03138733). We reported molecular characteristics of methicillin-resistant *S. aureus* (MRSA) along with the clinical outcomes.

**Figure:**
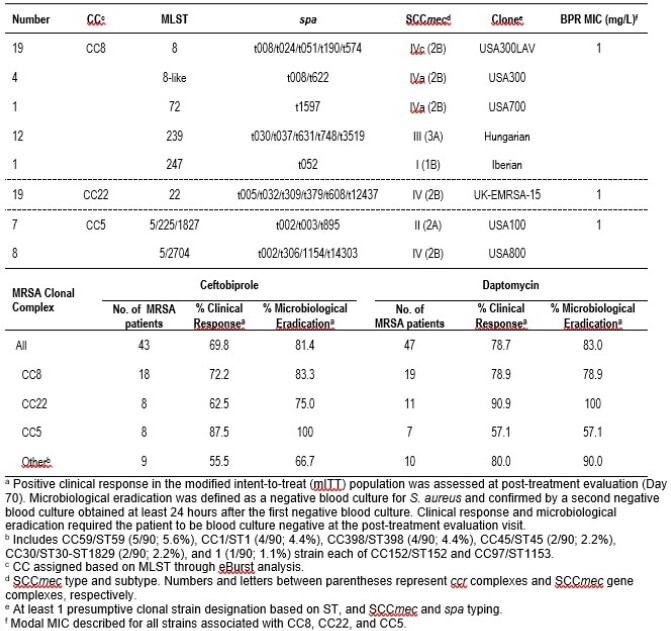

**Methods:**

94 patients had MRSA isolated from baseline blood cultures, 90 of which were available for molecular characterization. Susceptibility testing used the CLSI broth microdilution method. Total genomic DNA was extracted and sequenced. Assembled genomes were used to determine multilocus sequence typing (MLST), clonal complex (CC), *spa*, and SCC*mec* types.

**Results:**

A total of 10 CC were present within the 90 isolates. 71 (78.9%) MRSA fell into 3 predominant clones: CC8, CC22, and CC5 (Table). Among CC8 (37/90; 41.1%), 4 ST8-like (4/37; 10.8%) from USA (3) and Ukraine (1) were presumptively designated as USA300, whereas 12 ST239 (12/37; 32.4%) from Bulgaria (1), Georgia (1), Russia (1), Serbia (2), and Ukraine (7) were designated as the Hungarian clone. Other ST8-like MRSA (19/23; 82.6%) from Ukraine (15), Russia (2), Spain (1), and Argentina (1) were identified as USA300LAV. Nineteen (19/90; 21.1%) MRSA belonged to CC22/ST22. Fifteen (15/90; 16.7%) MRSA were CC5, and included ST5 and related single- (ST225 and ST2704) or double-locus (ST1827) variants. CC5 MRSA were designated as USA100 (7/15; 46.7%) or USA800 (8/15; 43.3%). Other strains were represented by 7 CC, including those associated with livestock infections. Overall, clinical responses (CR) and microbiological eradication (ME) were similar between treatment arms (Table). CR and ME rates were higher in the BPR arm among patients infected with CC5 strains, whereas these rates in the comparator arm were higher in patients infected with CC22 and other CC types.

**Conclusion:**

This global SAB trial included patients with MRSA strains belonging to pandemic lineages and those causing infections in United States hospitals. This strain set includes clones previously associated with hospital- and community-acquired infections as well as strains associated with livestock infections. In general, CR and ME were comparable between the 2 study arms with small differences in outcomes by CC type.

**Disclosures:**

**Rodrigo E. Mendes, PhD**, AbbVie: Grant/Research Support|Basilea: Grant/Research Support|Cipla: Grant/Research Support|Entasis: Grant/Research Support|GSK: Grant/Research Support|Paratek: Grant/Research Support|Pfizer: Grant/Research Support|Shionogi: Grant/Research Support **Leonard R. Duncan, PhD**, AbbVie: Grant/Research Support|Basilea: Grant/Research Support|CorMedix: Grant/Research Support|Melinta: Grant/Research Support|Pfizer: Grant/Research Support **John H. Kimbrough, PhD**, AbbVie: Grant/Research Support|Basilea: Grant/Research Support|Pfizer: Grant/Research Support|Shionogi: Grant/Research Support **Thomas L. Holland, MD**, Aridis: Advisor/Consultant|Basilea Pharmaceutica: Advisor/Consultant|Karius: Advisor/Consultant|Lysovant: Advisor/Consultant **Vance G. Fowler, MD, MHS**, Amphliphi Biosciences, Integrated Biotherapeutics; C3J, Armata, Valanbio; Akagera, Aridis, Roche, Astra Zeneca: Advisor/Consultant|Genentech, Regeneron, Deep Blue, Basilea, Janssen;: Grant/Research Support|Infectious Diseases Society of America: Honoraria|MedImmune, Allergan, Pfizer, Advanced Liquid Logics, Theravance, Novartis, Merck; Medical Biosurfaces; Locus; Affinergy; Contrafect; Karius;: Grant/Research Support|Novartis, Debiopharm, Genentech, Achaogen, Affinium, Medicines Co., MedImmune, Bayer, Basilea, Affinergy, Janssen, Contrafect, Regeneron, Destiny,: Advisor/Consultant|Sepsis diagnostic: Patent pending|UpToDate: Royalties|Valanbio and ArcBio: Stock Options **Mark E. Jones, PhD**, Astellas Pharma Global Development, Inc: Support for the present publication|Basilea Pharmaceutica International Ltd: Employee of Basilea Pharmaceutica International Ltd|Basilea Pharmaceutica International Ltd: Stocks/Bonds **Marc Engelhardt, MD**, Astellas Pharma Global Development, Inc.: Support for the present publication|Basilea Pharmaceutica International Ltd: Employee of Basilea Pharmaceutica International Ltd|Basilea Pharmaceutica International Ltd: Stocks/Bonds **Jennifer Smart, PhD**, Basilea Pharmaceutica International Ltd, Allschwil, Switzerland: Stocks/Bonds **Mariana Castanheira, PhD**, AbbVie: Grant/Research Support|Basilea: Grant/Research Support|bioMerieux: Grant/Research Support|Cipla: Grant/Research Support|CorMedix: Grant/Research Support|Entasis: Grant/Research Support|Melinta: Grant/Research Support|Paratek: Grant/Research Support|Pfizer: Grant/Research Support|Shionogi: Grant/Research Support

